# Spatiotemporal drought analysis in Bangladesh using the standardized precipitation index (SPI) and standardized precipitation evapotranspiration index (SPEI)

**DOI:** 10.1038/s41598-022-24146-0

**Published:** 2022-11-30

**Authors:** Mohammad Kamruzzaman, Mansour Almazroui, M. A. Salam, Md Anarul Haque Mondol, Md. Mizanur Rahman, Limon Deb, Palash Kumar Kundu, Md. Asad Uz Zaman, Abu Reza Md. Towfiqul Islam

**Affiliations:** 1 Farm Machinery and Postharvest Technology Division, Bangladesh Rice Research Institute, Gazipur, 1701 Bangladesh; 2 Centre of Excellence for Climate Change Research/Department of Meteorology, King Abdulaziz University, Jeddah, 21589 Saudi Arabia; 3 Climatic Research Unit, School of Environmental Sciences, University of East Anglia, Norwich, UK; 4 Agricultural Economics Division, Bangladesh Rice Research Institute, Gazipur, 1701 Bangladesh; 5 School of Earth, Atmosphere and Environment, Monash University, Clayton, VIC 3800 Australia; 6 Department of Geography and Environment, Jahangirnagar University, Savar, Dhaka, 1342 Bangladesh; 7 Irrigation and Water Management Division, Bangladesh Rice Research Institute, Gazipur, 1701 Bangladesh; 8 Rice Farming Systems Division, Bangladesh Rice Research Institute, 1701, Gazipur, Bangladesh; 9 Department of Disaster Management, Begum Rokeya University, Rangpur, 5400 Bangladesh

**Keywords:** Hydrology, Natural hazards

## Abstract

Countries depending on small-scale agriculture, such as Bangladesh, are susceptible to climate change and variability. Changes in the frequency and intensity of drought are a crucial aspect of this issue and the focus of this research. The goal of this work is to use SPI (standardized precipitation index) and SPEI (standardized precipitation evapotranspiration index) to investigate the differences in drought characteristics across different physiognomy types in Bangladesh and to highlight how drought characteristics change over time and spatial scales when considering different geomorphologies. This study used monthly precipitation and temperature data from 29 metrological stations for 39 years (1980–2018) for calculating SPI and SPEI values. To determine the significance of drought characteristic trends over different temporal and spatial scales, the modified Mann–Kendall trend test and multivariable linear regression (MLR) techniques were used. The results are as follows: (1) Overall, decreasing dry trend was found in Eastern hill regions, whereas an increasing drought trends were found in the in the rest of the regions in all time scaless (range is from − 0.08 decade^−1^ to − 0.15 decade^−1^ for 3-month time scale). However, except for the one-month time scale, the statistically significant trend was identified mostly in the north-central and northeast regions, indicating that drought patterns migrate from the northwest to the center region. (2) SPEI is anticipated to be better at capturing dry/wet cycles in more complex regions than SPI. (3) According to the MLR, longitude and maximum temperature can both influence precipitation. (4) Drought intensity increased gradually from the southern to the northern regions (1.26–1.56), and drought events occurred predominantly in the northwestern regions (27–30 times), indicating that drought meteorological hotspots were primarily concentrated in the Barind Tract and Tista River basin over time. Findings can be used to improve drought evaluation, hazard management, and application policymaking in Bangladesh. This has implications for agricultural catastrophe prevention and mitigation.

## Introduction

Drought is the most complicated of the recurrent extreme weather events since it is defined by a lack of rainfall over a considerable period of below-normal rainfall, and it can result in significant economic loss and human misery^1^. Droughts have become more frequent and severe in many regions of the world, including Bangladesh, as a result of global warming and climate change (increasing temperatures and shifting rainfall patterns)^2–4^. The average global temperature is expected to rise by 0.20 °C each decade for the rest of the twenty-first century at least, although the rate of growth will differ by area to area^5^. By the end of the twenty-first century, global temperatures are predicted to rise by 0.3–4.8 °C^6–8^. According to some research, Bangladesh is warming faster than the rest of the world ^7,9,10^, which is likely to lead to increased water demand and, as a result, a worsening of the country's drought.

Natural disasters are common in Bangladesh include floods, drought, cyclones, sea-level rise and salt intrusions. Currently, agriculture and water are two sectors that are seriously affected by the ongoing drought in the country. In the recent past, Bangladesh has been subjected to various degrees of drought, which have mostly damaged agricultural areas and resulted in enormous losses of food grains. Likewise, drought has an impact on the country's socio-economic environment and development efforts^11^. Bangladesh has had severe historical droughts in 1951, 1957, 1961, 1973, 1975, 1979, 1981, 1982, 1984, 1989, and 1995^11–13^, with the 1995 drought resulting in a net loss of 377,000 tons of Aman rice^14^. According to Ahmed^14^, 47% of the nation is at risk of drought, and 33% of the population lives in drought-prone regions. Drought reduced agricultural output by 25–30% in the northwestern region of Bangladesh in 2006, according to Habiba et. al.14. Despite the fact that drought planning and management in Bangladesh have received less attention than other hazards, drought is more devastating than floods and drought-related losses are larger than flood-related losses, according to research^11,16,17^. There has been a significant push to address drought management concerns, owing to the influence of climate change, the growing severity of drought occurrences, and human vulnerability. As a result, governments, scientists, and environmentalists are all working together to establish legislation that will help minimize the effects of drought.

To make matters worse, ecological over-exploitation of forest resources is causing soil erosion and harm to native plants, which in turn is causing the natural water cycle to become unbalanced. According to earlier studies, the main causes of drought are a lack of rainfall and an increase in temperature^18^. As a result, drought features at a specific location and time period may be assessed based on rainfall and temperature, and appropriate steps can be implemented to minimize drought frequency and intensity^19^.

The Drought Characteristic Index (DCI) is an indicator developed by the World Meteorological Organization (WMO) in 1992 and measures the cumulative effects and irregular water deficiencies of long-term drought conditions. It must be based on large-scale quantitative measurements over an extended time period and an accurate collection of historic records to be considered valid^20^.

The Palmer Drought Severity Index (PDSI) calculates the severity of any given drought by using climatologically appropriate precipitation as a proxy for water demand and assessing water loss based on definite precipitation discrepancies^21^. However, PDSI is spatially limited and hence cannot accurately explain large-scale drought changes^22^. Thomas B. McKee et al.^23^ advocated that the PDSI be replaced by the standardized precipitation index (SPI), which has a wide range of applications in terms of time, space, and rainfall probability distribution. It can detect drought features across a large area at different scales. Precipitation, the SPI discovered, is the most critical factor in influencing the intensity and duration of droughts^24^. An extension of the Standardized Precipitation Index (SPI) is the Standardized Precipitation Evapotranspiration Index (SPEI). For the purpose of determining drought, the SPEI considers both precipitation and potential evapotranspiration (PET). Since the SPI does not take into account the impact of rising temperatures on water demand, the SPEI does. It is possible to compute the SPEI over time frames ranging from one to forty-eight months, just like the SPI^25^. These drought indices have been used in various regions of the world to assess the presence of drought and examine its link to weather and climate^26–33^. Drought-prone areas can benefit from the combined usage of SPEI and SPI, which are widely used in current research.

The pre- and post-monsoon seasons are the most common periods for the occurrence of drought in Bangladesh. When monsoon rains begin later than expected, pre-drought conditions can linger throughout the monsoon season in extreme cases^34^. The extent to which a place is vulnerable to drought varies. It is predicted that precipitation will continue to increase in the majority of Bangladesh's areas, although it is projected to decrease in the southwest, according to one study^35^. Drought features from the past can be used to make predictions about future droughts^36^. As a result, it is critical to monitor the spatiotemporal characteristics of agricultural droughts in Bangladesh in order to anticipate adverse consequences and minimize potential losses and damages. Moreover, a drought monitoring and early warning system can help farmers and water managers plan for the most efficient use of water resources and maximize agricultural productivity.

Droughts occur as a result of climate change and a lack of soil moisture, resulting in reduced crop yields. One or more variables, such as large-scale downward air movement within the atmosphere or a deficiency of available moisture in the atmosphere, may be directly responsible for a lack of rain. However, elements affecting Bangladesh's rainfall have not yet been identified. As a first for the country of Bangladesh, a multivariate regression model of precipitation (Y) was developed using data from this study, which included variables such as elevation, maximum and minimum temperatures, longitude and latitude. The conditional probability was also utilized to evaluate the sensitivity of drought indices to better depict dry/wet alterations in more complicated locations.

Furthermore, the National Water Management Plan^37–39^ designated eight hydrological zones to plan the development of their water resources based on acceptable changes to their natural features. Southwest (SW), Northeast (NE), North Central (NC), Northwest (NW), South Central (SC), Southeast (SE), Eastern Hills (EH), and River and Estuary Region (RE) are the hydrological regions. Previously, no suitable activities in accordance with the Hydrological Region's drought risk management policy had been taken in Bangladesh.

This work has produced crucial information on changes in drought patterns, allowing researchers to learn more about anticipated future drought changes in Bangladesh. The article's primary research objectives include: (1) to compute the SPI and SPEI over a range of time scales using precipitation and evapotranspiration data; (2) Using the Mann–Kendall test (M–K trend test), to evaluate the drought trend of diverse geographical characteristics; (3) to undertake a spatiotemporal analysis of Bangladesh's drought hotspots by measuring drought intensity, drought frequency, and the precipitation trend coefficient; (4) to examine the elements that contribute to changes in precipitation trends across diverse topography using the multivariable linear regression (MLR) approach. The findings may serve as a solid foundation for the government to expand the diversity of drought-prevention zones in the future when environmental preservation is a priority. `

## Materials and methods

### Study area

**Figure 1:**
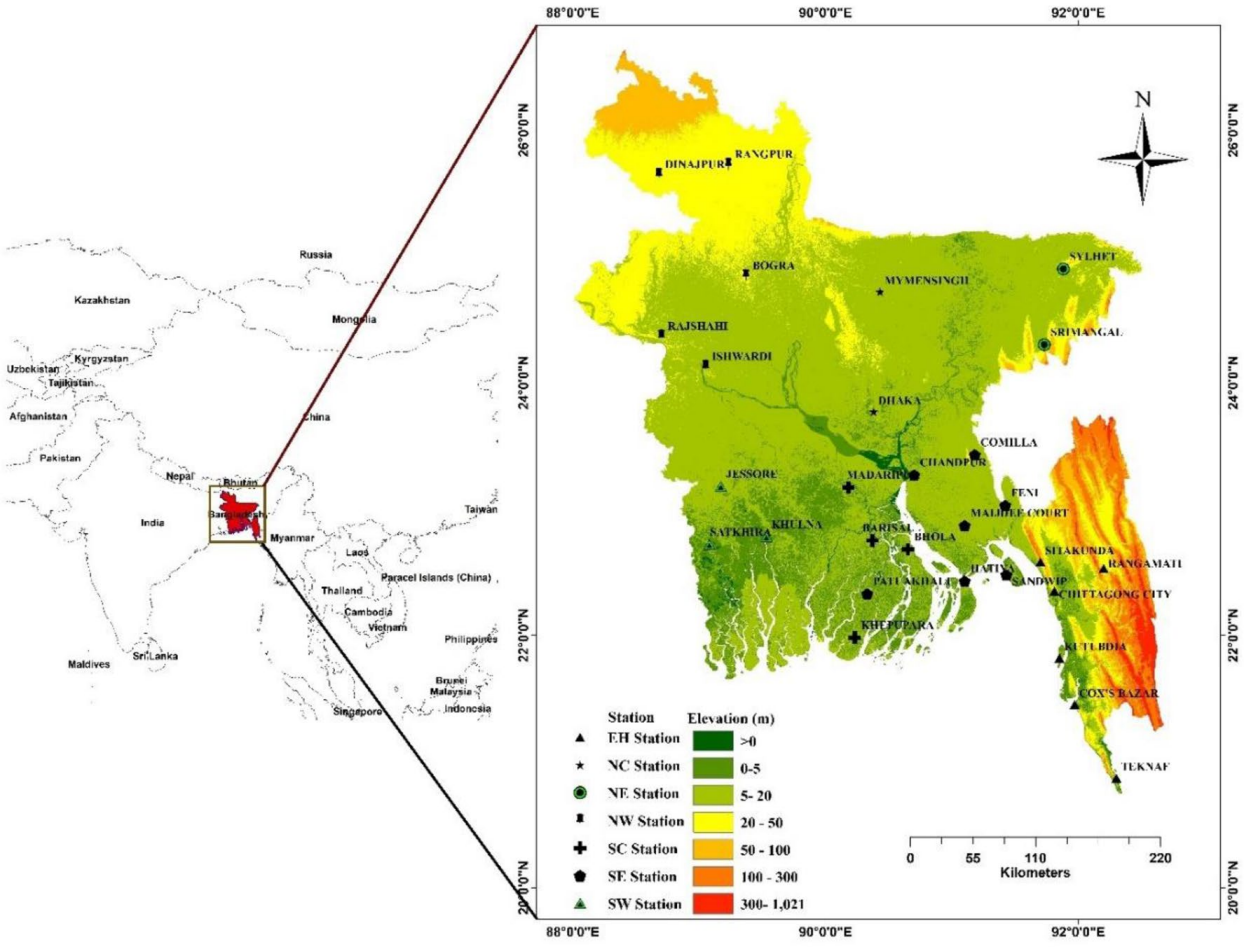
Hydrological regions of Bangladesh and the distribution of meteorological stations. (The vector and elevation data in this figure were obtained from Natural Earth (http://www.naturalearthdata.com) and the Shuttle Radar Topography Mission (SRTM) dataset of the United States Geological Survey (USGS) (http://eros.usgs.gov/), (The map is developed in Qgis 3.6 (https://www.qgis.org/en/site/)].

The study region includes all of Bangladesh and spans latitudes of 20°34′ to 26°38′ N and longitudes of 88°01′ to 92°41′ E in South Asia**.** In the west, north, and northeast, Bangladesh has borders with India; in the south, it shares a border with Myanmar. The Bay of Bengal, with its extensive coastline, marks the southern border (Fig. 1). The highest point in the country's northern region is 105 m above sea level, however, the rest of Bangladesh is no higher than 10 m.

Stations from the Bangladesh Meteorological Department (BMD) are now grouped into seven hydrological areas^37^ to study drought situations throughout the nation, as illustrated in Fig. 1, taking into account geography and land usage as well as anomalies in rainfall. The seven hydrological regions are referred to as: (i) Northwest (NW, 5 stations), (ii) North Central (NC, 2 stations), (iii) Northeast (NE, 2 stations), (iv) Southwest (SW, 3 stations), (v) South Central (SC, 4 stations), (vi) Southeast (SE, 7 stations), and (vii) Eastern Hills (EH, 6 stations).

The NW region covers the Rajshahi Administrative Division and its sixteen Districts, and is surrounded by the Brahmaputra and Ganges rivers. The average annual precipitation is approximately 1700 mm, however the south-western Barind Zone is one of the driest regions in Bangladesh, with annual precipitation below 1400 mm. High Barind is the only elevated ground in the region. The region is largely agriculturally developed. The NC region is surrounded to the north and northeast by the Brahmaputra, Padma, and Meghna rivers, as well as the Old Brahmaputra and Lakhya rivers. It is more diversified in terms of physiography than any other region except EH. The annual average precipitation ranges from 1400 to 2200 mm. Aside from the Madhupur Tract, agriculture resembles that of the Northwest region. The NE region is considerably wetter (3200 mm annual average precipitation), has relatively little accessible shallow groundwater except in the northwest due to aquifer issues, but has more dry season surface water resources. Aside from tea cultivation and natural gas extraction, the region is dominated by the 5600 km^2^ Haor Basin, which contains 47 major haors and 6300 beels, of which approximately 55% are perennial. The southwest (SW) region consists of two separate zones: the interior zone, which stretches from the Ganges and Padma rivers to roughly Khulna, and the coastal zone. The average annual precipitation is approximately 1700 mm, and along the Indian border in the west, there are areas with relatively little precipitation, similar to the Northwest. The SC Region, which is adjacent to the SW region and is surrounded by the Padma and Meghna rivers, has a similar inland–coastal zone distinction. Annual precipitation averages 2300 mm. In the south of the SC, numerous large tidal channels dissect the land area. Comilla, Brahmanbaria, Chandpur, Feni, Laxmipur, and Noakhali districts from SE regions. Like the SW and SC regions, this region has both an interior and a coastal zone. The Chittagong Coastal Plain and the Chittagong Hill Tracts (CHT) are the two distinct sub-regions that make up the EH region. The annual precipitation average is roughly 2400 mm.

Bangladesh is one of the countries that is most at risk from the escalating effects of climate change on a global scale. It is frequently struck by natural calamities, including flooding, drought, tornadoes, and tidal bores^40^. Bangladesh has recently faced drought on a frequent and regular basis; on average, at least once every 2.5 years^41^. Between 2011 and 2015, the frequency of droughts in all categories increased dramatically^1^. Despite the fact that drought is widespread in Bangladesh, the northwest region is particularly prone to it because of the region's high degree of rainfall variation^11^. Additionally, this region is relatively arid and characterized by sandy soils, receiving significantly less rainfall than the national average^12^. The moisture retention capacity of sandy soils is lower than that of other soil types, while infiltration is higher^15^. In addition, drought in the region has been exacerbated by the construction of the Farakka barrage in the Ganges River's upper reaches.

### Observation data

Because of the country's subtropical monsoon climate, temperatures, rainfall, and humidity all vary greatly from season to season throughout the year in Bangladesh. From March to May, there is a hot, humid summer; from June to September, there is a wet, warm, and rainy monsoon season; from October to November, there is autumn; and from December to February, there is a dry winter. In Bangladesh, three separate crop seasons exist Pre-Kharif (March–June), Kharif (July–October), and Rabi (November–February).

Bangladesh Meteorological Department (BMD) manages 35 meteorological stations throughout the country at the moment. Nonetheless, there are just 29 places with rainfall data going back more than 30 years. The 29 meteorological stations' monthly rainfall and temperature records from 1980 to 2018 were utilized to diagnose droughts. Missing data is a key issue when using observational data. Average values from neighboring stations were used to fill in missing data, which was around 2%. To obtain the spatial distribution of drought and pass the homogeneity test, we interpolated the data at a resolution of 1 km using inverse distance weighted (IDW) interpolation based on the placements of meteorological stations. Figure 1 depicts the research scope and the location of each site.

Between 1980 and 2018, there was a total of 2462.14 mm of rain recorded annually on the ground. Rainfall in Bangladesh peaks during the monsoon months (June to October) due to the weak humid depressions that are carried into Bangladesh by the moist monsoon winds^9^. One of the most notable aspects of Bangladesh's climate is the spatial and temporal unpredictability of the country's rainfall. Over the period 1980 to 2018, Bangladesh received rainfall ranging from approximately 1400 mm in the west to more than 4000 mm in the east. Meghalaya's elevation also contributes to the increased rainfall in the northeastern region.

### Calculation of standardized precipitation index (SPI)

Drought conditions in the research area can be estimated using the Standardized Precipitation Index (SPI). SPI is an index that uses solely precipitation data. This is based on the chance of precipitation for a few consecutive months, and its major aim is to reflect the deficiency of precipitation across an area on several time scales relative to its climatology^23^. In spite of the fact that the SPI approach is not a drought forecasting tool, the SPI methodology has been used to identify dry or wet conditions and analyze their influence on water resources management. SPI can be calculated at several time intervals, including 1, 3, 6, 12, and 24 months^42^.

Unless researchers have a firm grasp on the required intervals, this strong feature of drought can generate an overwhelming amount of data^43^. The SPI is calculated mathematically using the cumulative likelihood of a certain rainfall event occurring at a given station. To begin, a two-parameter gamma density distribution function is used to fit the precipitation frequency of a meteorological station for each calendar month. The function of the gamma distribution is presented.

1 $$f\left( x \right) = \frac{1}{{\beta^{\alpha } \Gamma \left( \alpha \right)}}x^{\alpha - 1} e^{ - x/\beta }$$

where *α* and *β* are the factors defining the shape and scale, respectively. Monthly precipitation is denoted by X. In fact, the main problem in the application of gamma distribution to hydrometeorological data is the estimation of two parameters: shape and scale. Generally, least square and moments are used to estimate the parameters in a statistical equation from a sample of data records. The THOM method has smaller variability and lower error compared to the least square and moments methods. It considers the physical lower bound of zero but no non-statistical upper bound using the maximum likelihood technique. The Thom method^44^ can be used to estimate the two parameters. Then, using the gamma cumulative distribution function, one may compute the cumulative probability G(x) at x. Finally, the inverse of the cumulative standard normal distribution function is used to turn G(x) into the SPI value. Lue et al.^45^ introduced a thorough computation of SPI and drought categorization in their study.

### Calculation of standardized precipitation evapotranspiration index (SPEI)

SPEI is based on SPI but includes a temperature component to account for the effect of temperature on drought development through a basic water balance calculation, the difference between precipitation and reference evapotranspiration (P–ET_0_), rather than precipitation (P) as the input. The climatic water balance contrasts the available water (P) with the atmospheric evaporative demand (ET_0_) and, as a result, provides a more reliable indicator of drought severity than examining precipitation alone. The initial formulation of the SPEI recommended estimating ET_0_ using the Thornthwaite (Th) equation (Thornthwaite, 1948). This equation requires simply the monthly average temperature and latitude of the site, and it was utilized due to the scarcity of data.

The SPEI intensity scale calculates both positive and negative values to distinguish between wet and dry situations. It can be calculated for time steps ranging from one month to more than 48 months. Monthly updates enable for operational use, and the longer the available time series of data, the more reliable the results will be.

To determine the value of SPEI, the difference in the water balance is normalized as a log-logistic probability distribution. The probability density function can be expressed using the following equation:

2 $$f\left( x \right) = \frac{\beta }{\alpha }\left( {\frac{x - \gamma }{\alpha }} \right)\left[ {1 + \left( {\frac{x - \gamma }{\alpha }} \right)} \right]^{ - 2}$$

where the parameters scale, shape, and origin are denoted by *α*, *β*, and $\gamma$, respectively. Thus, the probability distribution function can be described in terms of a probability density function.

3 $$F\left( x \right) = \left[ {1 + \left( {\frac{\alpha }{x - \gamma }} \right)^{\beta } } \right]^{ - 1}$$

Vicente-Serrano et al.^42^ defined the SPEI as follows:

4 $$SPEI = W - \frac{{C_{0} + C_{1} W + C_{2} W^{2} }}{{1 + d_{1} W + d_{2} W^{2} + d_{3} W^{3} }}$$

When *P* ≤ *0.5*, $W = \sqrt { - 2ln\left( P \right)}$ and when *P* > 0.5, $W = \sqrt { - 2ln\left( {1 - P} \right)}$, C_0_ = 2.5155, C_1_ = 0.8028, C_2_ = 0.0203, d_1_ = 1.4327, d_2_ = 0.1892, d_3_ = 0.0013.

Using the SPEI package for R^46^, the SPI and SPEI drought index were calculated for this article. It's a great research and practical tool for assessing drought conditions. SPI/SPEI values were used to classify the severity of the drought, as shown in Table 1. SPI/SPEI values with a reduction in rainfall are indicative of drought, while SPEI values with a rise in rainfall are indicative of wetter or more typical conditions.

**Table 1:** Classification of drought based on SPI/SPEI values.

SPEI and SPI values	Drought category
0.99 to − 0.99	Normal
− 1.0 to − 1.49	Moderate drought
− 1.5 to − 1.99	Severe drought
≤ − 2	Extreme drought

#### Runs theory

Drought characteristics include the duration of the drought, its severity, and its frequency. SPI/SPEI is under normal conditions (SPI/SPEI ≥  − 1) is also calculated from the general technique when the absolute value of SPI/SPEI is calculated, which has a substantial impact on drought assessment. Thus, we employed Yevjevich’s run theory^47^ to define the severity and frequency of droughts. The run theory constructs a segment of the drought variable time series with all values less than or larger than the set threshold. This segment is referred to as a negative or positive run. The formula for calculating drought intensity is:

5 $$S = \frac{{\mathop \sum \nolimits_{n = 1}^{T} \left| {S_{SPI/SPEI} - K} \right|}}{T}$$

where drought intensity is denoted by S, $S_{SPI/SPEI}$ denotes an SPI or SPEI value less than or equal to the drought threshold, K denotes drought threshold, which in this study is set to be less than or equal to − 1, indicating that the severity of the drought is greater than that of moderate drought, and T is the duration of the drought.

Drought frequency is a metric for determining how often a drought occurs in a certain area, and its formula is as follows:

6 $$DF = \frac{n}{N} \times 100$$

where N is the time period during which the site was detected, and n denotes the number of droughts that occurred at the site during that time period.

### Conditional probability

The term "conditional probability" refers to the likelihood of occurrence of a particular event A in the presence of another event B, i.e., Cp (A/B). However, the Cp (SPI) in this study refers to the possibility of an of SPEI_drought_ occurring during an SPI_drought_ and vice versa for Cp(SPEI). The formula is as follows:

7 $$Cp \left( {SPI} \right) = \frac{{T_{SPI/SPEI} }}{{T_{SPEI} }}\,\,{\text{or}}\,\,Cp \left( {SPEI} \right) = \frac{{T_{SPEI/SPI} }}{{T_{SPI} }}$$

whereas T_SPI_ and T_SPEI_ reflect drought periods in a certain location over time depending on the SPI/SPEI value, T _SPI/SPEI_ and T_SPEI/SPI_ indicate the times of droughts in an area based on the SPEI/SPI evaluation that drought has happened, while SPI/SPEI conducts a re-evaluation of the region's drought.

The primary reason for using conditional probability distributions in this research is that conditional properties can provide drought probability distributions under various conditions. The conditional return periods of drought severity, peak, and duration are primarily based on a copula-based conditional probability distribution. Copulas can be used to incorporate precipitation and drought indexes and provide conditional probability distributions between them (with precipitation as the condition). We can calculate the likelihood of drought occurrence under various precipitation conditions using this conditional probability distribution. We can employ conditional probabilities to assess how prior information affects drought probabilities. In this study, a conditional probability distribution based on copulas was applied to meteorological drought analysis.

### Trend test

The Modified Mann–Kendall test (MMK) was introduced by Hamed and Rao^48^, to address the issue of serial correlation by the use of the variance correction approach. When applied to autocorrelation time series data, nonparametric trend tests such as Mann–Kendall produce incorrect or excessive rejection rates^41^. Time series data autocorrelation within a time series, also recognized as serial dependency, is widely regarded as one of the most difficult problems in time series data analysis and trend detection. To address autocorrelation issues in time series data, the modified Mann–Kendall trend test is used. For computing, the MK test the modified variance (Var(S)) is applied^49,50^ and the subsequent Eqs. (8)–(10) are applied to compute the autocorrelation:

8 $${\text{Var}}\left( {\text{S}} \right)* = {\text{Var}}\left( {\text{S}} \right) \times \left( {\frac{{\text{n}}}{{\text{n*}}}} \right)$$

9 $$\left( {\frac{{\text{n}}}{{\text{n*}}}} \right) = 1 + \left( {\frac{2}{{{\text{n}}\left( {{\text{n}} - 1} \right)\left( {{\text{n}} - 2} \right)}}} \right) \times \mathop \sum \limits_{{{\text{k}} = 1}}^{{{\text{n}} - 1}} \left( {{\text{n}} - {\text{k}}} \right)\left( {{\text{n}} - {\text{k}} - 1} \right)\left( {{\text{n}} - {\text{k}} - 2} \right)^{{\text{r}}} \cdot {\text{k}}$$

10 $${\text{r}}_{{{\text{k }} = }} \frac{{\left( {\frac{1}{{{\text{n}} - {\text{k}}}}} \right)\mathop \sum \nolimits_{{{\text{i}} = 1}}^{{{\text{n}} - {\text{k}}}} \left( {{\text{x}}_{{\text{i}}} - {\text{x}}} \right)\left( {{\text{x}}_{{{\text{i}} + {\text{k}}}} - {\text{x}}} \right)}}{{\left( {\frac{1}{{\text{n}}}} \right)\mathop \sum \nolimits_{{{\text{i}} = 1}}^{{\text{n}}} { }({\text{x}}_{{\text{i}}} - {\text{x}})^{2} { }}}$$

where nn* and ${\text{r}}_{{\text{k }}}$ implies the modified coefficient of autocorrelated data and autocorrelation coefficient of k-th lag, respectively; x denotes the mean of the time series. The significance of the trend at a 95% confidence interval of the k-th lag can be estimated by Eq. (11):

11 $$\left( {\frac{{ - 1 - 1.96\sqrt {{\text{n}} = {\text{k}} - 1} }}{{{\text{n}} - {\text{k}}}}} \right) \le {\text{r}}_{{\text{k }}} \left( {95{\text{\% }}} \right) \le \left( {\frac{{ - 1 + 1.96\sqrt {{\text{n}} = {\text{k}} - 1} }}{{{\text{n}} - {\text{k}}}}} \right)$$

The 95% confidence level is achieved if the ${\text{r}}_{{\text{k }}}$ satisfies the upper condition. Hence, the dependence of the data and influence of the autocorrelation between different time lags should be eliminated for estimating the trend.

In this study, we used the MMK test to detect drought spatiotemporal trends. The ability to eliminate the influence of autocorrelation on test significance is the main advantage of using the MMK test instead of the MK test. The traditional MK test does not take autocorrelation into account, which is common in hydroclimatological time series. Positive autocorrelation increases the likelihood of testing significance and vice-versa for negative autocorrelation^48^.

### Sen’s slope estimator

The nonparametric Sen’s slope (SS) technique^51^ was employed in this study to estimate the rate of trend magnitude in time-series datasets, this method was used to determine the magnitude of a trend. In comparison to other methods, the impact of an outlier on-trend outcomes is negligible using this strategy^52^. The Sen’s slope (SS) can be calculated by Eq. (12)

12 $$\beta = Median\left[ {\frac{{x_{j} - x_{i} }}{j - i}} \right] all j > i$$

where x_j_ denotes the jth values and x_i_ the ith values in observational data. A positive value of β denotes an increase whereas a negative value indicates a decreasing rate of change.

### Multivariable linear regression method

Multivariable linear regression is a type of regression that includes both linear and nonlinear regressions with multiple explanatory variables. The method of multivariable linear regression (MLR) is used to construct a multiple regression model in which meteorological variables affect their geographical interpolation components. We use precipitation (Y) as the dependent variable and altitude (H), maximum temperature (Tmax), minimum temperature (Tmim), longitude (Lo), and latitude (La) as the independent variables.. There are two major benefits to using an MLR model to analyze data. The ability to determine the relative influence of one or more predictor variables on the criterion value is the first. The ability to identify outliers, or anomalies, is the second advantage. The MLR model was developed to address the dependence structure of the characteristics to increase the efficiency of the estimates in hydrometeorological studies. Despite its advantages, it includes a lengthy and complicated process of computation. We assume that each independent variable has a linear effect on the dependent variable and that the mean value of precipitation changes evenly when one independent variable changes while the others remain constant. The present study built a multiple regression model of precipitation (Y) for altitude (H), maximum temperature (Tmax), minimum temperature (Tmin), longitude (Lo), and latitude (La), and estimated the residuals using the following expression:

13 $$Y\left( {H, Tmax,Tmin, Lo, La} \right) = b0 + b1H + b2Tmax + b3Tmin + b4Lo + b5La + \varepsilon$$

where b1, b2, b3, b4, b5 are the unknown coefficients; b0 is a constant and ɛ is the residual value^45^.

## Results

### Patterns of drought on a multi-scale

Monthly SPI and SPEI values were computed for 29 weather stations from 1980 to 2018 at five unique time scales (1, 3, 6, 12, and 24 months), as depicted in Fig. S1a–g. SPI and SPEI values were then averaged across five timescales to define drought conditions in Bangladesh. Wet/dry transitions are more obvious as the time scale shortens, while the sensitivity of SPI and SPEI measurements varies substantially.

To examine the overall trends in SPEI and SPI in Bangladesh, the non-parametric MM–K test was employed to investigate drought trends across many locations and time scales from 1980 to 2018. (Table 2). The MM–K test reveals a downward trend in the SPEI value for Bangladesh's drought-prone NW area, whereas the SPI value shows an upward trend over several time scales (Table 2). According to the trend statistics, drought conditions continued to deteriorate across the country from 1980 to 2018, except for the Eastern Hilly (EH) region. When SPI/SPEI was estimated with more lagged time scales, the SPEI downward trend was larger than that of SPI, and the drought trend was steadily growing.

**Table 2:** MK Trend test coefficient and significant coefficient (P) of SPI and SPEI on various time scales in different hydrologic regions of Bangladesh.

Region	SPEI	Trend (decade^−1^ )	Z value	*P*-value	SPI	Trend (decade^−1^ )	Z -value	*P* value
	Scale				Scale			
EH	SPEI1	− 0.04	− 1.23	0.22	SPI1	0.03	0.77	0.44
	SPEI3	0.00	0.05	0.96	SPI3	0.07	1.28	0.20
	SPEI6	0.07	1.04	0.30	SPI6	0.14	1.86	0.06
	SPEI9	0.12	1.74	0.08	SPI9	0.23	2.59	**0.01**
	SPEI12	0.17	2.37	**0.02**	SPI12	0.26	3.17	**0.02**
	SPEI24	0.20	2.32	**0.02**	SPI24	0.30	3.27	**0.01**
	SPEI1	− 0.06	− 1.13	0.26	SPI1	− 0.10	− 2.21	**0.03**
	SPEI3	− 0.15	− 2.03	**0.04**	SPI3	− 0.18	− 2.37	**0.02**
NC	SPEI6	− 0.19	− 2.11	**0.04**	SPI6	− 0.20	− 2.25	**0.02**
	SPEI9	− 0.22	− 2.40	**0.02**	SPI9	− 0.22	− 2.39	**0.02**
	SPEI12	− 0.26	− 2.51	**0.01**	SPI12	− 0.26	− 2.69	**0.01**
	SPEI24	− 0.35	− 2.61	**0.01**	SPI24	− 0.36	− 2.80	**0.01**
	SPEI1	− 0.06	− 1.13	0.26	SPI1	− 0.10	− 2.21	**0.03**
	SPEI3	− 0.15	− 2.03	**0.04**	SPI3	− 0.18	− 2.36	**0.02**
NE	SPEI6	− 0.19	− 2.11	**0.04**	SPI6	− 0.20	− 2.25	**0.02**
	SPEI9	− 0.22	− 2.40	**0.02**	SPI9	− 0.22	− 2.39	**0.02**
	SPEI12	− 0.26	− 2.51	**0.01**	SPI12	− 0.26	− 2.69	**0.01**
	SPEI24	− 0.35	− 2.61	**0.01**	SPI24	− 0.36	− 2.80	**0.01**
	SPEI1	− 0.05	− 1.23	0.22	SPI1	− 0.02	− 0.55	0.57
	SPEI3	− 0.08	− 1.19	0.24	SPI3	0.00	− 0.02	0.98
NW	SPEI6	− 0.09	− 0.97	0.33	SPI6	0.03	0.43	0.66
	SPEI9	− 0.07	− 0.82	0.41	SPI9	0.09	1.18	0.23
	SPEI12	− 0.09	− 1.14	0.26	SPI12	0.10	1.16	0.24
	SPEI24	− 0.13	− 1.60	0.11	SPI24	0.05	0.82	0.41
	SPEI1	− 0.04	− 0.87	0.38	SPI1	− 0.0	− 0.87	0.38
	SPEI3	− 0.06	− 0.80	0.43	SPI3	− 0.05	− 0.70	0.48
SC	SPEI6	− 0.05	− 0.87	0.38	SPI6	− 0.04	− 0.53	0.59
	SPEI9	− 0.03	− 0.44	0.66	SPI9	− 0.09	0.00	1.00
	SPEI12	− 0.04	− 0.44	0.66	SPI12	− 0.02	− 0.16	0.86
	SPEI24	− 0.06	− 0.58	0.56	SPI24	− 0.01	− 0.12	0.90
	SPEI1	− 0.05	− 1.23	0.22	SPI1	− 0.02	− 0.55	0.57
	SPEI3	− 0.08	− 1.19	0.24	SPI3	0.00	− 0.02	0.98
SE	SPEI6	− 0.09	− 0.97	0.33	SPI6	0.03	0.43	0.66
	SPEI9	− 0.07	− 0.82	0.41	SPI9	0.09	1.18	0.23
	SPEI12	− 0.09	− 1.14	0.26	SPI12	0.10	1.16	0.24
	SPEI24	− 0.13	− 1.60	0.11	SPI24	0.05	0.82	0.41
	SPEI1	− 0.06	− 1.60	0.11	SPI1	− 0.05	− 1.18	0.23
	SPEI3	− 0.07	− 1.02	0.31	SPI3	− 0.06	− 1.24	0.21
SW	SPEI6	− 0.08	− 1.05	0.29	SPI6	− 0.06	− 0.73	0.46
	SPEI9	− 0.03	− 0.35	0.73	SPI9	0.03	0.29	0.77
	SPEI12	0.00	− 0.04	0.97	SPI12	0.06	0.56	0.56
	SPEI24	− 0.08	− 0.81	0.42	SPI24	0.00	0.00	1.00

*NE* North East, *NC* North Central, *NW* North West, *SC* South Central, *SW* South West, *SE* South East, and *EH* Eastern Hill.

Significance values are in bold.

On all time periods, the SPEI drought trend in the NC and NE regions was larger than that in the SW, SE, SC, and NW regions (− 0.15 decade^−1^ for 3-month in NC and NE, − 0.06 decade^−1^ for 3-month in SC, − 0.07 decade^−1^ for 3-month in SW, − 0.08 decade^−1^ for 3-month in NW and SE), indicating that the degree of drought in central Bangladesh is growing and the drought trend is gradually changing from southwest to east.

SPEI is more responsive to drought fluctuations than SPI since it incorporates evapotranspiration. The difference between SPI and SPEI was determined using conditional probability (Cp). Table 3 summarizes the Cp (SPI) and Cp (SPEI) values for various areas at multiple time scales. Cp (SPI) was 0.54,0.52,0.44 and Cp (SPEI) was 0.46, 0.48,0.56,0 in the southern coastal areas from 1980 to 2010 at 3- month time scale, and the difference in the results was not statistically significant (*P* > 0.05), indicating SPI and SPEI can be used together to validate drought conditions at a certain time in a certain area. In some areas and throughout particular time periods, however, SPI and SPEI exhibit vastly different characteristics of drought than one another (*P* < 0.05). For instance, from 1980 to 2018, Cp (SPI) dropped continually whereas Cp (SPEI) climbed consistently (Cp (SPI) was 0.56,0.21,0.43,0.14 and Cp (SPEI) was 0.44,0.79,0.57, 0.86 on a 1-month time scale from 1980 to 2018. Cp (SPEI) was greater than Cp (SPI) on the time scale over all regions. Dry/wet cycles in more complex regions are likely to be captured with greater accuracy by SPEI than by SPI, according to these findings.

**Table 3:** The conditional probability of SPI and SPEI in different regions of Bangladesh throughout various time periods and time scales [Cp (SPI) and Cp (SPEI)]. (‘/’ indicates that no drought exists, and hence the conditional probability cannot be calculated).

Region	Period	Cp (SPI)						Cp (SPEI)					
		1	3	6	9	12	24	1	3	6	9	12	24
EH	1980–1990	0.47	0.64	0.68	0.67	0.70	0.71	0.53	0.36	0.32	0.33	0.30	0.29
	1991–2000	0.13	0.42	0.43	0.42	0.44	0.26	0.28	0.58	0.57	0.58	0.56	0.74
	2001–2010	0.19	0.43	0.40	0.50	0.40	0.33	0.47	0.57	0.60	0.50	0.60	0.67
	2011–2018	0.09	0.44	0.44	0.33	0.00	0.50	0.31	0.56	0.56	0.67	0.99	0.50
NC	1980–1990	0.33	0.40	0.27	0.50	0.50	/	0.67	0.60	0.73	0.50	0.50	/
	1991–2000	0.67	0.49	0.48	0.50	0.48	0.33	0.94	0.51	0.52	0.50	0.53	0.67
	2001–2010	0.67	0.59	0.50	0.50	0.44	0.56	0.78	0.41	0.50	0.50	0.56	0.44
	2011–2018	0.61	0.49	0.46	0.45	0.49	0.50	0.89	0.51	0.54	0.55	0.51	0.50
NE	1980–1990	0.39	0.48	0.47	0.50	0.44	0.40	0.61	0.52	0.53	0.50	0.56	0.60
	1991–2000	0.30	0.47	0.42	0.45	0.49	0.43	0.70	0.53	0.58	0.55	0.51	0.58
	2001–2010	0.38	0.46	0.50	0.50	0.41	0.44	0.62	0.54	0.50	0.50	0.59	0.56
	2011–2018	0.76	0.41	0.48	0.41	0.42	0.00	0.24	0.59	0.52	0.59	0.58	0.99
NW	1980–1990	0.56	0.36	0.45	0.33	0.60	/	0.44	0.64	0.55	0.67	0.40	/
	1991–2000	0.21	0.46	0.44	0.46	0.50	0.44	0.79	0.54	0.56	0.54	0.50	0.56
	2001–2010	0.43	0.60	0.46	0.50	0.50	0.50	0.57	0.40	0.54	0.50	0.50	0.50
	2011–2018	0.14	0.48	0.50	0.49	0.49	0.43	0.86	0.52	0.50	0.51	0.51	0.57
SC	1980–1990	0.30	0.54	0.58	0.69	0.68	0.80	0.70	0.46	0.42	0.31	0.32	0.20
	1991–2000	0.40	0.50	0.43	0.48	0.47	/	0.60	0.50	0.57	0.52	0.53	/
	2001–2010	0.41	0.50	0.48	0.46	0.52	0.40	0.59	0.50	0.52	0.54	0.48	0.60
	2011–2018	0.33	0.42	0.43	0.48	0.47	0.48	0.67	0.58	0.57	0.52	0.53	0.52
SE	1980–1990	0.55	0.54	0.65	0.70	0.64	0.75	0.45	0.46	0.35	0.30	0.36	0.25
	1991–2000	0.47	0.52	0.57	0.55	0.47	0.67	0.53	0.48	0.43	0.45	0.53	0.33
	2001–2010	0.40	0.44	0.38	0.57	0.64	0.22	0.60	0.56	0.62	0.43	0.36	0.78
	2011–2018	0.27	0.35	0.40	0.00	0.00	/	0.73	0.65	0.60	0.99	0.99	0.99
SW	1980–1990	0.37	0.48	0.53	0.54	0.47	0.63	0.63	0.52	0.47	0.46	0.53	0.38
	1991–2000	0.45	0.42	0.45	0.50	0.50	0.40	0.55	0.58	0.55	0.50	0.50	0.60
	2001–2010	0.42	0.50	0.42	0.50	0.46	0.33	0.58	0.50	0.58	0.50	0.54	0.67
	2011–2018	0.40	0.50	0.49	0.45	0.47	0.39	0.60	0.50	0.51	0.55	0.53	0.61

*NE* North East, *NC* North Central, *NW* North West, *SC* South Central, *SW* South West, *SE* South East, and *EH* Eastern Hill.

### Precipitation trends and factors affecting precipitation over Bangladesh

**Figure 2:**
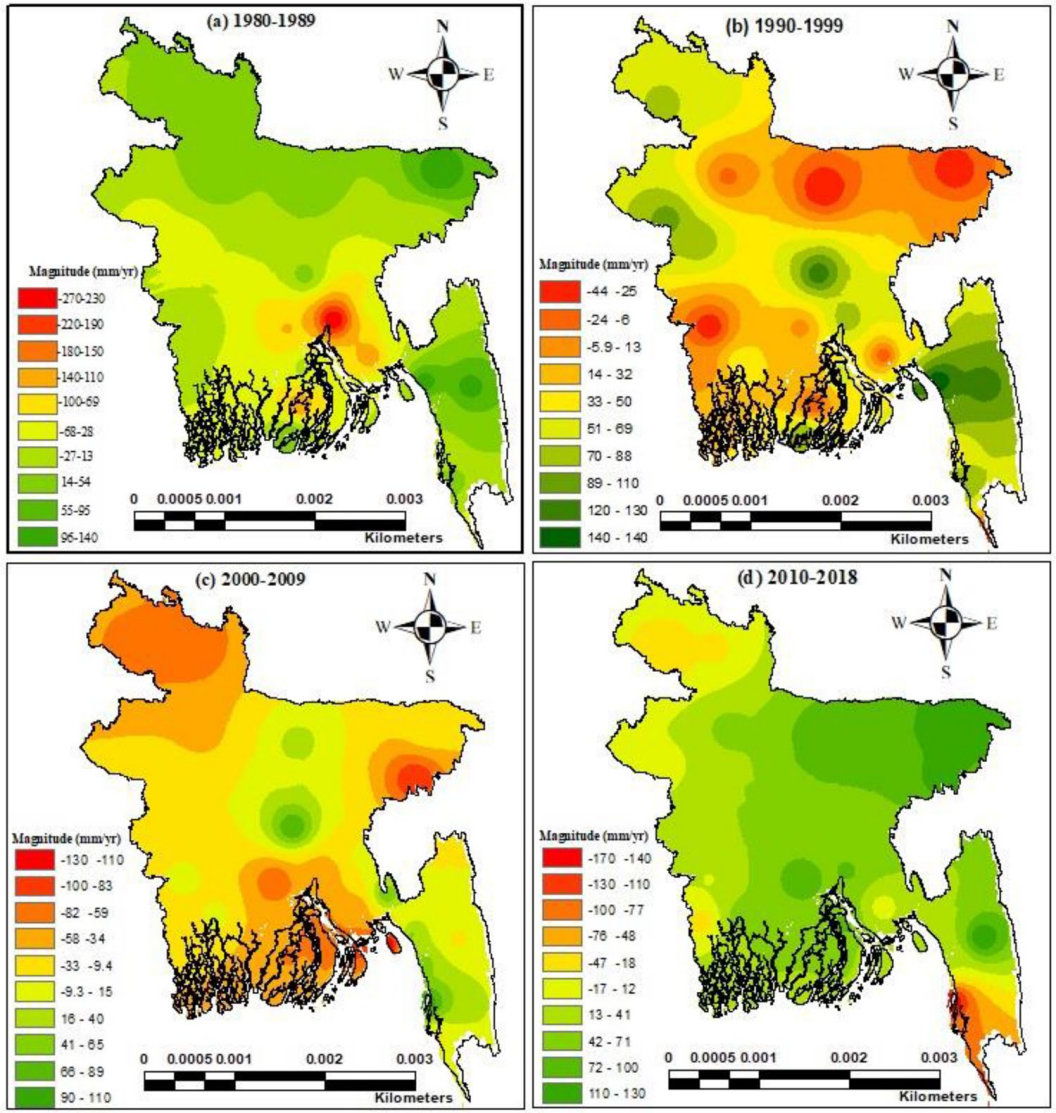
The MK Trend test coefficient for Bangladesh's precipitation throughout a range of time periods (1980–1989s, 1990–1999s, 2000–2009s, and 2010–2018s). [The figure was gebnerated by R software (R version 4.2.1) (https://cran.r-project.org/bin/windows/base/)].

To conduct a secondary analysis of the trend in Bangladesh's drought characteristics from 1980 to 2018, as seen in Fig. 2, we studied the meteorological changes at 29 stations in Bangladesh over the past four decades (1980–1989, 1990–1999, 2000–1910, and 2011–2018) using the nonparametric MMK test considering precipitation data.

Between 1980 and1989s, 69% of stations demonstrated decreasing precipitation trends, although only 10% of the sites demonstrated significance (*P* < 0.05), with the magnitude of the trend ranging from – 273.21 to 136.33 mm year^−1^. Between 1990 and 1999, rainfall increased in the majority of the country (76% of stations) except for seven stations, with the magnitude of the trend ranging from − 10 to 144 mm year^−1^. However, the increasing/decreasing trends are all insignificant (*P* > 0.5). The negative trend was dominant in the north-eastern region of Bangladesh, while parts of the southwestern region have begun to exhibit negative tendencies. In contrast, decreasing rainfall trends were found in most of the country (76% of stations) except for seven stations, during the years 2000–2009. The magnitude of the trend ranged from − 132 to 114 mm year^−1^. However, those trends are not significant (*P* > 0.5). Between 2010 and 2010, the majority of sites in Bangladesh showed positive trends, while just 27.5% showed negative trends. The sites with the largest magnitude trends (166.27 mm year^-1^) from 1990 to 2018 were in eastern hilly regions.

The multivariable linear regression (MLR) method was used to develop a multiple regression model of average annual precipitation over various time periods based on altitude (H), maximum temperature (Tmax), minimum temperature (Tmin), longitude (Lo), and latitude (La), as well as to determine the model's significance coefficients (Table 4). The findings reveal that the annual precipitation average for each period is significantly affected by the maximum temperature and longitude. Precipitation, maximum temperature, and longitude all demonstrate negative trends significantly (*P* < 0.05), indicating that precipitation declines with increasing longitude and maximum temperature.

**Table 4:** Regression equation relating geographical and climatic factors (elevation (H), maximum temperature (Tmax), minimum temperature (Tmin), longitude (Lo), and latitude (La)) to annual precipitation at meteorological stations across time.

Period	Regression equation	R^2^	P(H)	P(T_max_)	P(T_min_)	P(L_o_)	P(L_a_)
1981–1990	Y = − 7.37 × H – 346.7 × T_max_ + 50.0 × T_min_ + 474.4 × L_o_ + 143.8 × L_a_ − 34,240.4	0.49	**0.02**	**0.00**	0.49	**0.00**	**0.03**
1991–2000	Y = 2.37 × H – 263.9 × T_max_ + 165.5 × T_min_ + 421.6 × L_o_ − 45.9 × L_a_ − 30,122.9	0.52	0.46	**0.00**	**0.01**	**0.00**	0.43
2001–2010	Y = − 1.80 × H – 381.4 × T_max_ − 73.1 × T_min_ + 384.2 × L_o_ − 188.5 × L_a_ − 14,616.2	0.49	0.57	**0.00**	0.31	**0.00**	**0.00**
2011–2018	Y = − 4.55 × H – 288.3 × T_max_ + 123.3 × T_min_ + 588.3 × L_o_ − 65.5 × L_a_ − 42,926.6	0.59	0.23	**0.00**	0.09	**0.00**	0.30

P(H), P(Tmax), P(Tmin), P(Lo), and P(La) represent the t-test result; a value less than 0.05 indicates that the variable is statistically significant.

Significance values are in bold.

### Assessment of drought frequency and intensity using SPEIs in different zones

Drought characteristics include frequency, duration, severity and intensity. Therefore, we applied multiple timescale SPEIs to run theory, and analyzed the changes in drought frequency and intensity from 1980 to 2018 at different decadal time sacles. Table 5 shows these characteristics using the threshold value (SPEI =  − 1.0).

**Table 5:** Drought intensity and frequency using SPEI at different time scales.

Zone	Period	Drought frequency*						Drought intensity*					
		SPEI-1	SPEI-3	SPEI-6	SPEI-9	SPEI-12	SPEI-24	SPEI-1	SPEI-3	SPEI-6	SPEI-9	SPEI-12	SPEI-24
EH	1981–1990	16	13	10	10	7	12	1.24	1.18	1.23	1.19	1.20	1.09
	1991–2000	10	12	13	15	14	15	1.37	1.44	1.34	1.34	1.29	1.13
	2001–2010	16	17	9	5	3	8	1.32	1.22	1.25	1.29	1.05	1.08
	2011–2018	11	11	5	2	2	1	1.16	1.22	1.33	1.10	1.17	1.17
NC	1980–1990	12	8	8	3	1	0	1.36	1.23	1.16	1.09	1.11	1.10
	1991–2000	17	21	24	22	21	12	1.40	1.43	1.39	1.43	1.44	1.16
	2001–2010	15	11	10	6	7	2	1.24	1.32	1.23	1.19	1.11	1.04
	2011–2018	17	22	31	35	40	50	1.20	1.30	1.29	1.28	1.32	1.52
NE	1980–1990	10	11	10	6	5	3	1.38	1.34	1.19	1.27	1.34	1.15
	1991–2000	21	17	26	29	31	23	1.28	1.41	1.31	1.34	1.31	1.33
	2001–2010	14	17	8	8	10	6	1.26	1.24	1.38	1.27	1.19	1.28
	2011–2018	20	22	15	11	11	13	1.24	1.31	1.35	1.14	1.16	1.18
	1980–1990	10	11	10	6	5	3	1.38	1.24	1.11	1.12	1.10	1.15
NW	1980–1990	11	6	6	4	3	0	1.31	1.36	1.35	1.36	1.44	1.23
	1991–2000	15	15	17	19	17	5	1.11	1.22	1.24	1.18	1.22	1.01
	2001–2010	5	10	8	10	9	1	1.19	1.31	1.25	1.21	1.23	1.23
	2011–2018	22	18	21	26	26	49	1.27	1.19	1.12	1.19	1.14	1.17
SC	1980–1990	15	11	15	10	12	3	1.32	1.37	1.32	1.35	1.22	1.15
	1991–2000	11	12	13	12	10	0	1.14	1.34	1.24	1.11	1.19	1.12
	2001–2010	17	11	10	7	3	2	1.19	1.31	1.27	1.28	1.26	1.26
	2011–2018	18	23	24	28	34	34	1.31	1.16	1.28	1.20	1.16	1.06
SE	1980–1990	9	11	9	9	8	3	1.23	1.28	1.33	1.28	1.23	1.01
	1991–2000	8	15	12	14	12	1	1.15	1.21	1.18	1.26	1.13	1.22
	2001–2010	16	16	9	3	3	2	1.28	1.24	1.19	1.09	1.19	1.10
	2011–2018	9	15	11	6	4	9	1.41	1.25	1.22	1.28	1.23	1.49
SW	1980–1990	16	13	15	16	22	4	1.34	1.32	1.20	1.30	1.26	1.21
	1991–2000	12	16	19	18	25	11	1.29	1.33	1.45	1.51	1.26	1.12
	2001–2010	9	14	8	4	2	3	1.33	1.36	1.35	1.32	1.34	1.35
	2011–2018	14	22	27	32	31	25	1.24	1.18	1.23	1.19	1.20	1.09

*Threshold value for identifying a drought is − 1.0 for SPEI.

Drought frequency increases due to climate change from 1980 to 2018. The SPEIs indicated that the frequency of three-month drought is greater than that of the other time scales. In particular, drought frequency identified with the three-month SPEI in 2011–2018 was 2/3 times greater than in 1980–1990 except for the EH region. Therefore, climate change could cause more severe and frequent droughts in the future in most of the regions of Bangladesh.

During 1980s (1980–1990), according to SPEI-1, drought zones with an intensity greater than 1.4 were largely situated in SW region of Bangladesh. Nevertheless, throughout the 1990s (1991–2000), drought intensity values larger than 1.4 were seen across the nation with the exception of the southern coastal regions (SC, SE, and SW) and were more prevalent in the NC and NW regions based on different time scales. In the 2000s, drought intensity was found to be weaker than in previous decades, with values less than 1.4 in most regions, with the exception of the southwestern regions. From 2010 to 2018, the drought territory with an intensity of more than 1.4 shifted from the southern coastal regions to the NC region.

### Spatial distridution of drougth intensity and frequency

**Figure 3:**
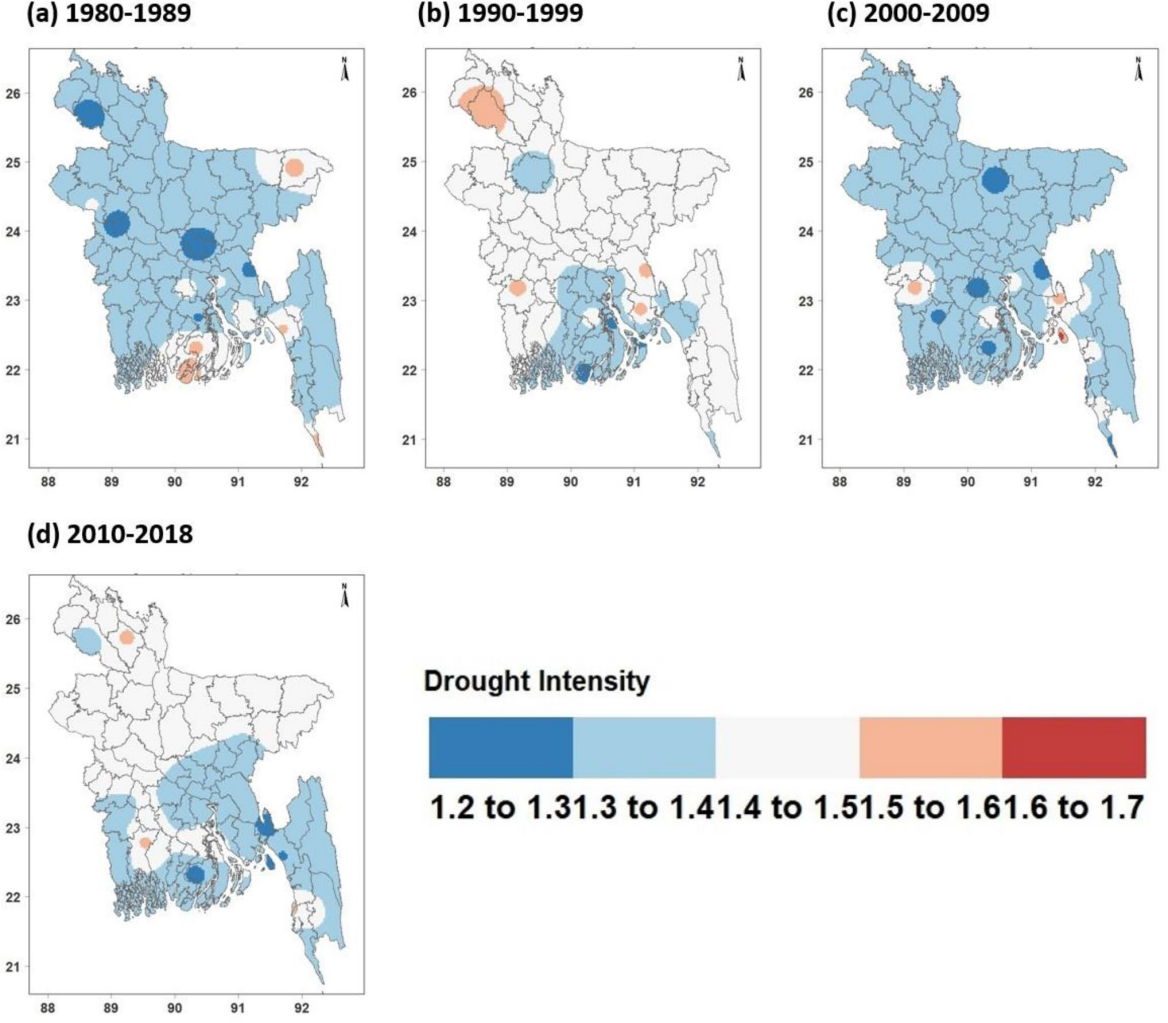
Based on 3-month SPEI, drought intensity during individual decades (1980–1989s, 1990–1999s, 2000–2009s, 2010–2018s) in Bangladesh. [The figure was gebnerated by R software (R version 4.2.1) (https://cran.r-project.org/bin/windows/base/)].

**Figure 4:**
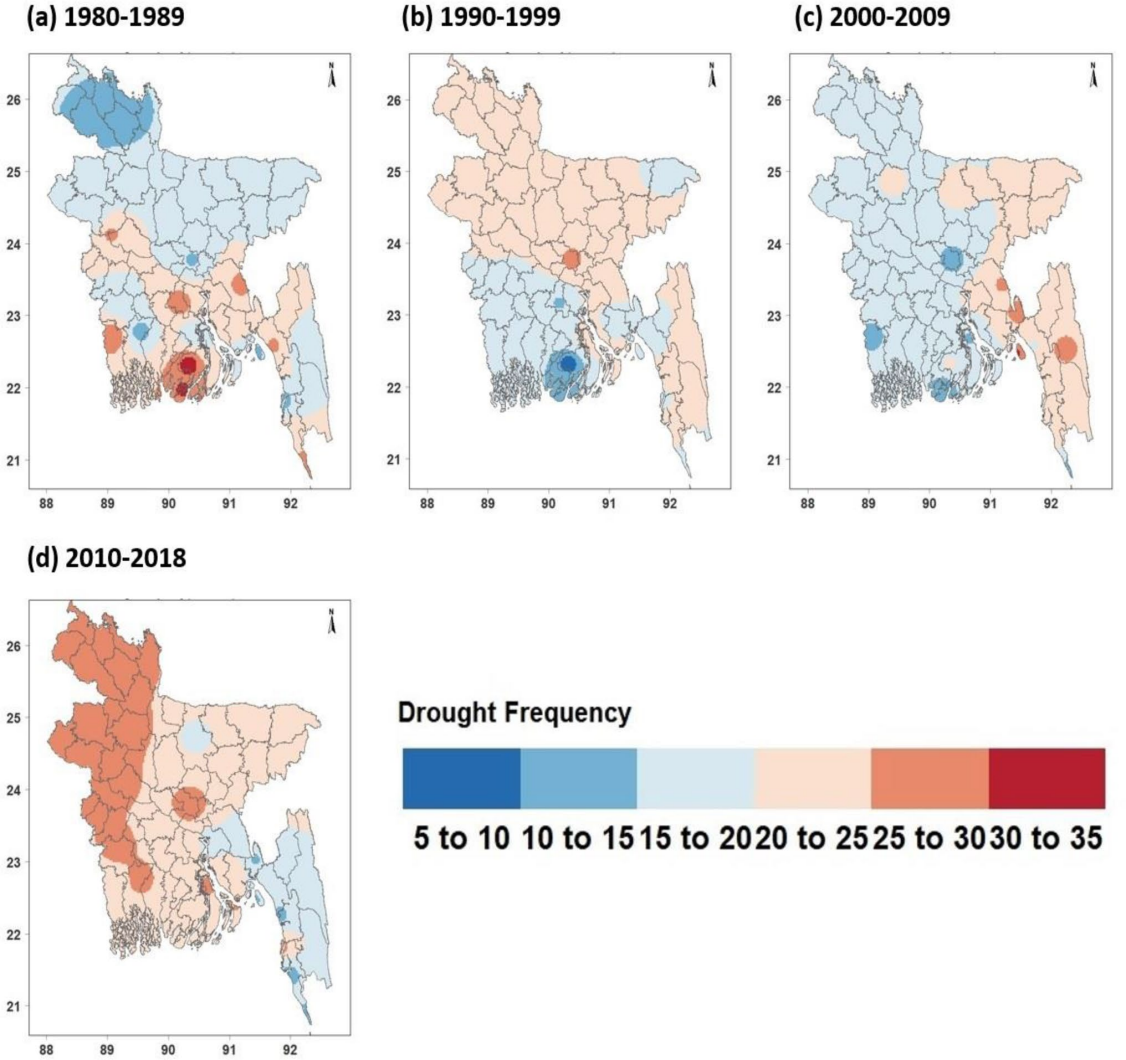
Based on 3-month SPEI, drought frequency during individual decades (1980–1989s, 1990–1999s, 2000–2009s, 2010–2018s) in Bangladesh. [The figure was gebnerated by R software (R version 4.2.1) (https://cran.r-project.org/bin/windows/base/)].

In terms of defining the temporal and spatial variability of drought, SPEI was more sensitive to drought assessment than SPI. According to Kamruzzaman et al.^54^, 3-month time scale is better able to detect drought events in Bangladesh than any other. Since SPEI was found to be a good indication of changes in drought, the run theory and 3-month SPEI were used to create a spatial distribution map for the inter-annual fluctuation of drought intensity and frequency (Figs. 3 and 4).

According to Fig. 3, drought areas with an intensity of more than 1.5 were primarily located in the northeast region and the southern coastal region of Bangladesh during the 1980s. However, in 1990s, drought intensity values greater than 1.4 were found all over the country except for the coastal region and were dominant in the northwest region. In the 2000s the drought intensity was found to be weaker than in other decades, with values less than 1.4 over most regions. However, from 2010 to 2018, the drought territory with an intensity of more than 1.4 changed from the southern coastal regions to the western part of the country.

We used 3-month SPEI values from 29 meteorological stations in Bangladesh from 1980 to 2018 to identify drought meteorological hotspots based on drought frequency, calculating the frequency of moderate drought and above at each station (Fig. 4).

The findings indicate that from 1980 to 2018, the frequency of droughts exceeding moderate levels was between 3 and 4% at each of Bangladesh's 29 meteorological stations, implying that the majority of the country typically experiences a drought event every two years. Between 1980 and 1989, drought frequency was highest at Patuakhali and Khepupara stations, which were physically located in the southeast and south regions, respectively, with frequencies of 34 and 31 events, during the period. Between 1990 and 1999, the drought frequency reduced in the majority of Bangladesh; even in the southeast, a drought occurred just 6 times.

However, the frequency of drought in Bangladesh dropped dramatically between 2000 and 2009. Droughts occurred primarily in the southeast and northeastern regions, particularly in locations such as Swandip, Feni, and Rangamati, where droughts happened 30, 28, and 27 times, respectively. Between 1990 and 2010, the eastern hilly region's drought frequency decreased to 13–19 times, except in Kutubdia, whereas drought frequency increased dramatically over the remaining regions. The greatest rise was observed in the northwest and southwest regions, where droughts occurred between 27 and 30 times.

## Discussion

Our findings confirmed prior findings that the frequency of droughts has increased in the northwest and southwest areas of Bangladesh^10,11,55–59^. Three main factors, including erratic spatiotemporal rainfall behaviour, geographic position, and high temperature, can lead to a rise in the frequency of drought, all of which is supported by the existing literature^50,60–62^. Prodhan et al.^60^ used statistical tools to monitor drought status in Bangladesh and discovered that the drought trend in the northwest and southwest regions has gradually increased over the last decades, while the drought trend in the southeast and northeast regions has steadily decreased over the last decade. Climate change and warming are the leading causes for the diversification of drought phenomena^10^. Additionally, the country's monsoon rainfall pattern is influenced by an irregular atmospheric circulation pattern known as the Southern Oscillation (SO), which reflects a relationship with EL Nino, or the quasi-periodic warming of sea surface temperature (SST)^50^ in a phenomenon known collectively as El Nino-Southern Oscillation (ENSO). Low rainfall occurred during the years of moderate El Nino (2002–2003 and 2009–2010) and strong El Nino (2014–2016)^63,64^. The long-term dry phase increases as SST are closely associated with the rainfall pattern^58,65^. Bangladesh is also influenced by regional topography, with the Bay of Bengal in the south and the Mighty Himalayas in the north having a significant effect on rainfall and drought patterns in the country.

Our study found that the monthly maximum temperature is inversely correlated with longitude over a lengthy period of time. Simultaneously, Bangladesh's average temperature distribution is much longer than its latitude zonality. The monthly average temperature has been negatively correlated with altitude for many years, indicating that temperature, latitude, longitude, and altitude are the primary factors affecting drought and rainfall. Furthermore, the elements triggered by Bangladesh's internal geographic location and atmospheric circulation have an indirect relationship with irregular weather and climate occurrences. Rahman et al.^66^ discovered that active convective inputs in the Indian Ocean's subtropical zone can induce sinking airflow over the southwest continental region, limit precipitation, and cause temperature anomalies.

In Bangladesh, the uneven spatial distribution of rainfall is determined by atmospheric conditions and is impacted by longitude and geographic position^67^. The method of multivariable linear regression (MLR) was used to create a multiple regression model of mean annual precipitation on various timescales based on altitude (H), maximum temperature (Tmax), minimum temperature (Tmin), longitude (Lo), and latitude (La), and to compute its significance coefficients (Table 4). The findings revealed that longitude and maximum temperature could notably affect the mean annual rainfall at each timescale. Precipitation, maximum temperature, and longitude exhibited significant negative trends (*P* < 0.05), implying that higher longitude and temperature triggered less rainfall. This study highlights that the coefficient of influence of temperature and longitude on precipitation is lower than altitude. Thus, altitude is not a critical contributing factor for the spatial disparity of rainfall in the country; multiple geographic factors determine the rainfall. Based on the results of the regression analysis, annual precipitation drops by 1.80–7.37 mm for every 100 m of altitude rise, and yearly rainfall declines by 263.9–346.7 mm for each degree of temperature increase, while the factor with the highest impact is the change in longitude, under which the more northwest and western part of the country receives less annual precipitation.

According to the MMK test, we computed the trend variations of SPEI of various climatic zones on multiple periods and found that all climatic zones exhibited negative trends from 1980 to 2018, except for the eastern hill regions. The trend coefficient of northcentral and northeastern parts is also higher than that of the remaining area, indicating that the degree of drought in the northcentral and northeastern parts of the country has steadily intensified. The drought trend gradually shifted from the southwest to the east. In contrast, the precipitation trend showed positive trends transfer from the northwest to the east and converge in the southeastern region. Earlier drought studies on the western part of Bangladesh have revealed that most of the regional droughts have increased notably in intensity and duration, particularly in the northwest part of the country, while for the moderate drought category, the trend coefficient from west to east in the northeastern part is declining^11,55,57^. Thus, it is crucial to explore the distribution of drought characteristics in Bangladesh on a regional scale.

The pattern of drought frequency has undergone notable geographic position changes; the distribution of drought frequency is more in the northwest and southwest and less in the south and east. The primary cause is the low-lying floodplain in the northwest part. Less rainfall and higher maximum temperature lead to increased evaporation capacity, and the foothills of the mountains affect the highland thermodynamic vertical circulation process, with the development of an atmospheric circulation backdrop consisting of little rainfall and many drought events. Though the degree of drought in the country's central zone continues to rise, we observed that the northwest area is already a drought-prone zone on various time scales from 1980 to 2018. The drought hotspots are primarily in the northwest part, particularly in the Barind Tract and Tista River basins. The reason for meteorological hotspots is that these regions are surrounded by the foothills of the Himalayan Mountain, making it hard for the southwest monsoon to release its moisture after the air in this region sinks, triggering this region to become a meteorological hotspot with recurrent drought events. This outcome is consistent with earlier works^64,68,69^. For example, Hossain et al. and Shahid et al.^11,70^ showed that the frequency and intensity of moderate drought events have increased in the northwest region. However, Abedin et al., Abdullah and Islam et al.^57,71,72^ disagreed with the current finding. They found that more moderate droughts occurred in southern and southwestern parts of the country compared to northwest parts. The distribution of drought characteristics over various periods suggests that a targeted drought control scheme, including strengthening the reservoir infrastructure facility regionally, developing rainwater harvesting tools, popularizing agricultural water-saving technology locality, and altering agricultural transplanting structure periodically, can be helpful for drought mitigation and adaption.

The findings of the SPEI outweighed the SPI over long periods, indicating that temperature was also a driving factor for drought occurrence as well as the mere absence of precipitation. This was true primarily for meteorological (3-month) and agricultural droughts. Our results exhibited agreement with previous studies of Homdee et al. and Fung et al.^73,74^. The main difference between SPI and SPEI is the extra meteorological factors applied to compute the SPEI indices, such as P and P-PET, respectively. SPI mainly considers climatic water supply parameters, while SPEI considers both climatic water demand and supply^61,75^. Consistent conclusions were found with earlier cited works using the SPEI which underlined the importance of climatic water demand and supply in drought studies^76–78^. Although precipitation is a crucial driver of meteorological drought, the rising temperature can significantly influence meteorological drought severity^42^. Therefore, SPEI is a comparatively better indicator than SPI under climate change because it is susceptible to temperature variation.

Our research establishes a systematic framework for drought monitoring, evaluation, and risk management in Bangladesh, as well as providing guidance and potential for policy implementation. SPI and SPEI are both meteorological drought indicators that take into account a variety of factors such as precipitation, evaporation, and temperature, and have a wide range of applicability for determining regional drought status. Due to the many perspectives on and interests in drought monitoring from the weather, agricultural, and water conservancy sectors, SPI and SPEI are proving to be not entirely adequate for addressing the actual needs of the different stakeholders. in real-time drought monitoring. Thus, drought monitoring remains a long-term and critical task.

## Conclusions

On multiple time and space scales, this study analyzed the meteorological and geographical drought characteristics in Bangladesh using SPEI, SPI, drought precipitation trend, drought intensity, and drought frequency. The following are the major conclusions:

1. On all time scales, the SPEI drought trend in the NC and NE regions was greater than that in the SW, SE, SC, and NW regions (− 0.015 year^−1^ for 3-month in NC and NE, − 0.006 year^−1^ for 3-month in SC, − 0.007 year^−1^ for 3-month in SW, − 0.008 year^−1^ for 3-month in NW and SE). This indicates that the central part of Bangladesh is becoming more drought-prone, and the drought trend is moving from the southwest to the east.
2. In specific places and time periods, there are considerable discrepancies in the drought characteristics between SPI and SPEI; Cp (SPI) decreases uninterruptedly while Cp (SPEI) rises continuously from 1980 to 2018. Moreover, Cp (SPEI) was larger than Cp (SPI) on all time scales, indicating that SPEI was more sensitive to drought assessment than SPI.
3. The correlation coefficient between altitude and rainfall is lower than the correlation coefficient between maximum temperature and longitude, demonstrating that height is not the primary factor driving the regional unevenness of precipitation in Bangladesh (MLR). According to the correlations, differences in geographical location and maximum temperature are most likely to be the cause of erratic rainfall.
4. We discovered that the intensity of drought in Bangladesh's eastern hilly region gradually decreased over time, while the intensity of drought in the southern region gradually increased to the northern (NW and SW) region (1.26–1.56), while drought events occurred primarily in the northwestern regions (27–30 times), indicating that drought meteorological hotspots were primarily concentrated in the Barind Tract and Tista River basin over time.

## Supplementary Information

Supplementary Figures.

## Data Availability

The datasets used and/or analysed during the current study available from the corresponding author on reasonable request.
