# Supplementary material for: Spatiotemporal drought analysis in Bangladesh using the standardized precipitation index (SPI) and standardized precipitation evapotranspiration index (SPEI)
Source: Sci Rep. 2022 Nov 30;12:20694. doi: 10.1038/s41598-022-24146-0 (PMC9712418; doi:10.1038/s41598-022-24146-0)
Supplement: Supplementary file 1 — Supplementary Figures. [file 41598_2022_24146_MOESM1_ESM.docx]

**Supplementary Information**

| **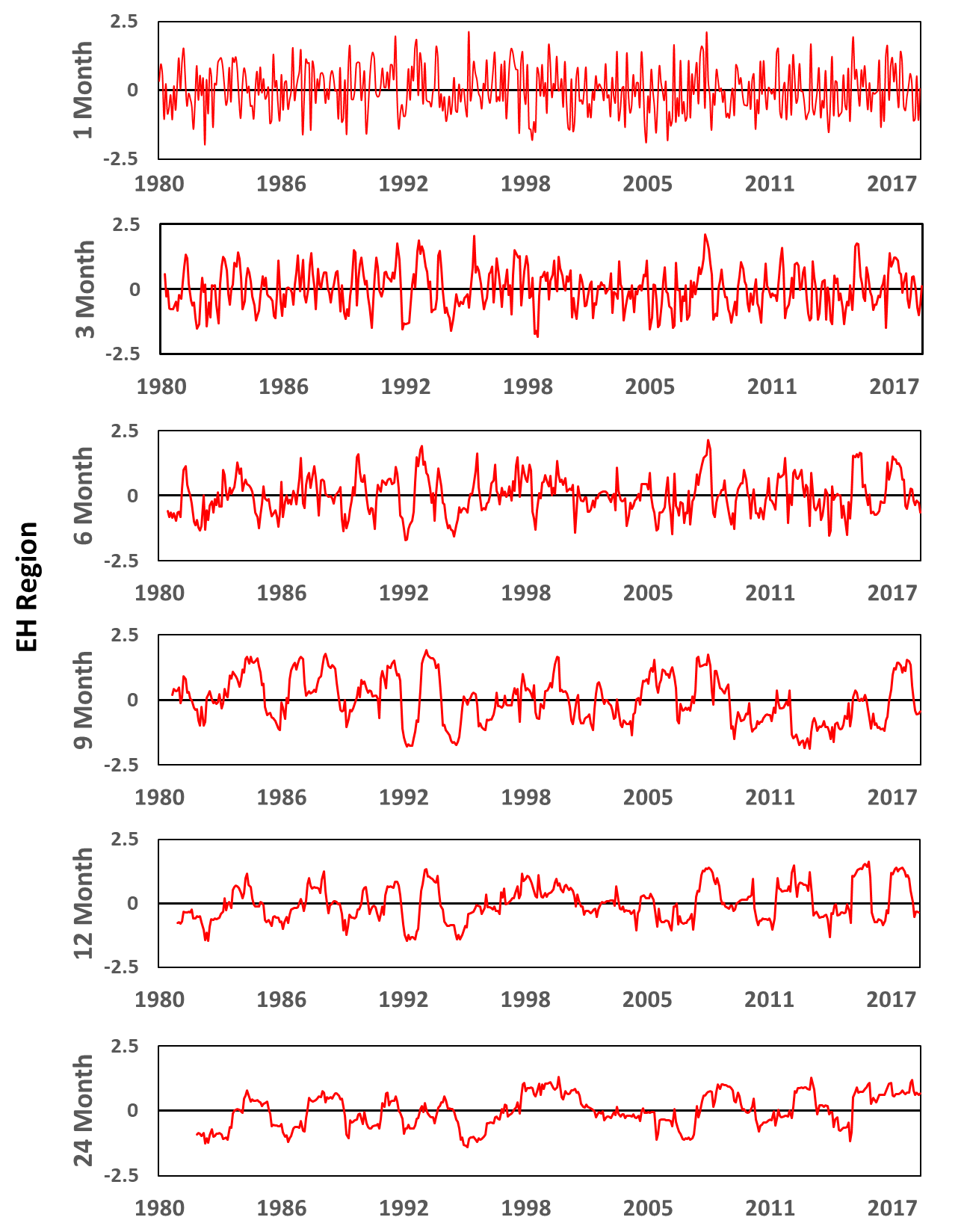** | **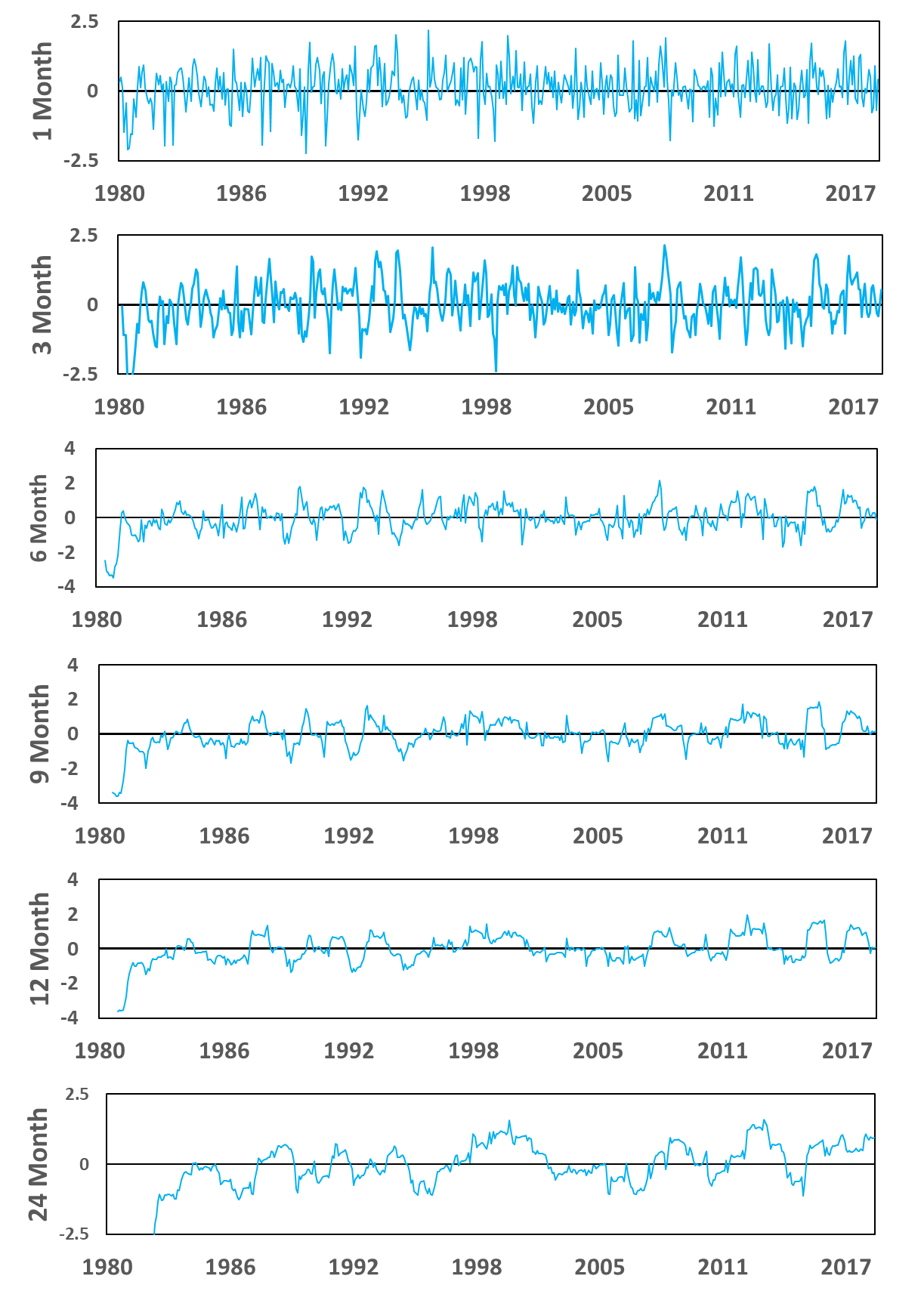** |
| --- | --- |

**Figure S1 (a) The SPI (left side red colour and SPEI (right side blue colour) values at different timescales for Eastern Hills (EH) region**

| **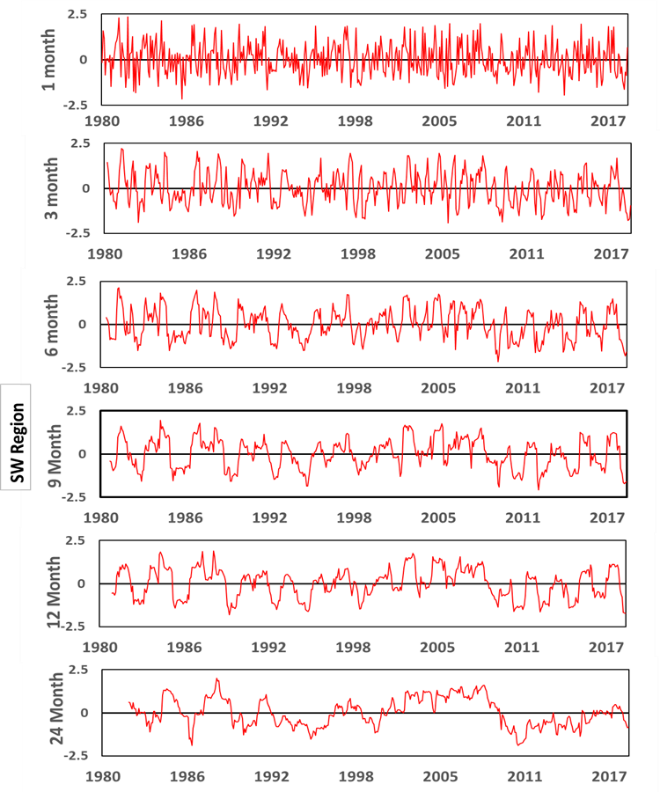** | **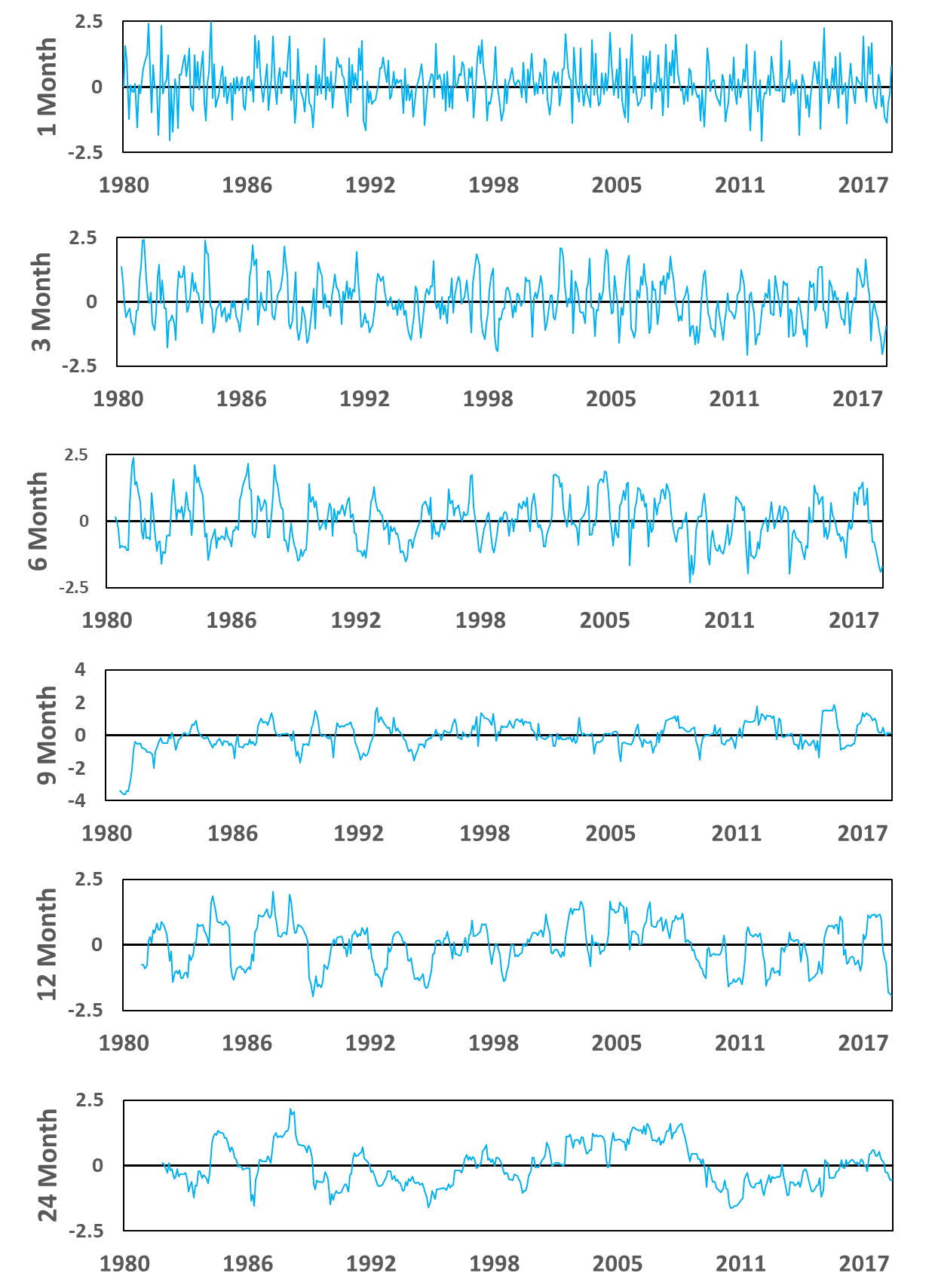** |
| --- | --- |

**Figure S1 (b) Same as Figure S1 (a), but for Southwest (SW) region**

| **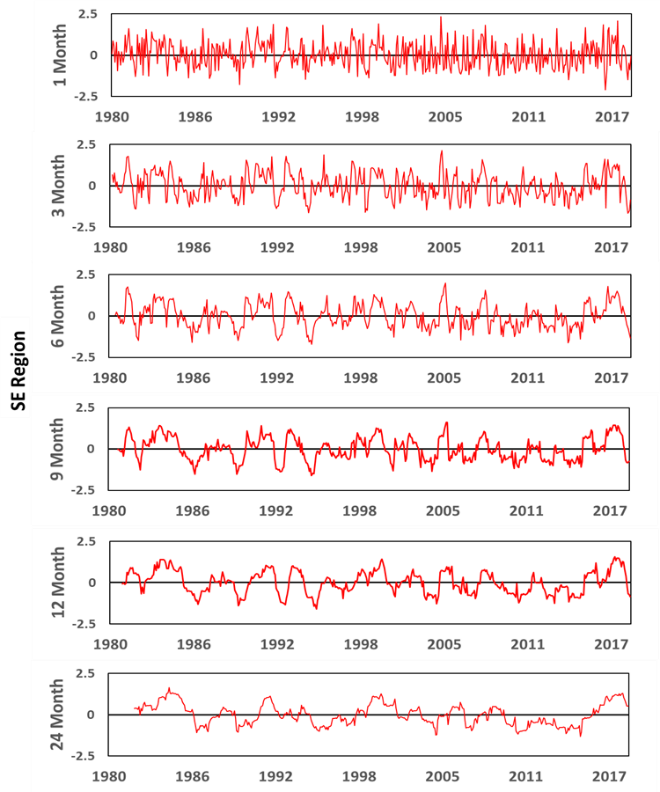** | **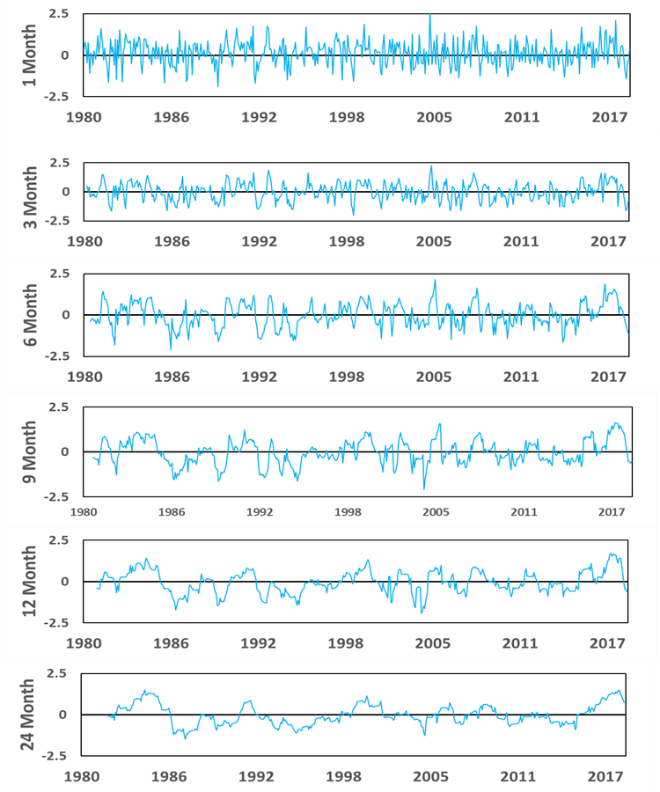** |
| --- | --- |

**Figure S1 (c) Same as Figure S1 (a), but for Southeast (SE) region**

| **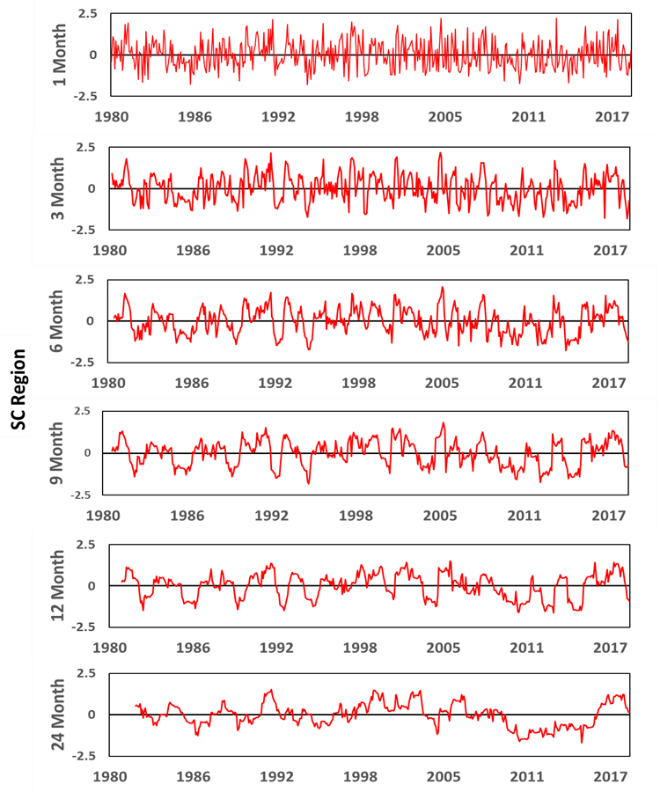** | **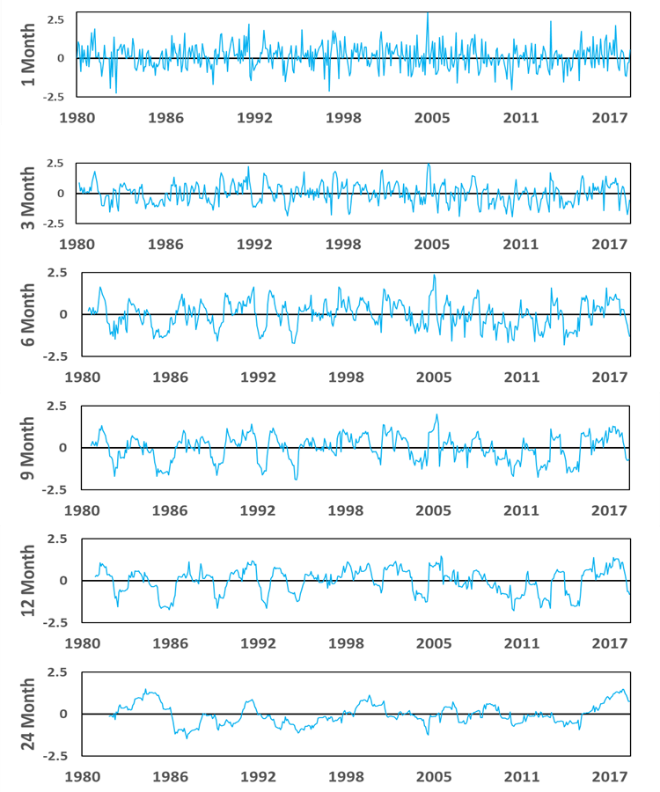** |
| --- | --- |

**Figure S1 (d) Same as Figure S1 (a), but for South Central (SC) region**

| **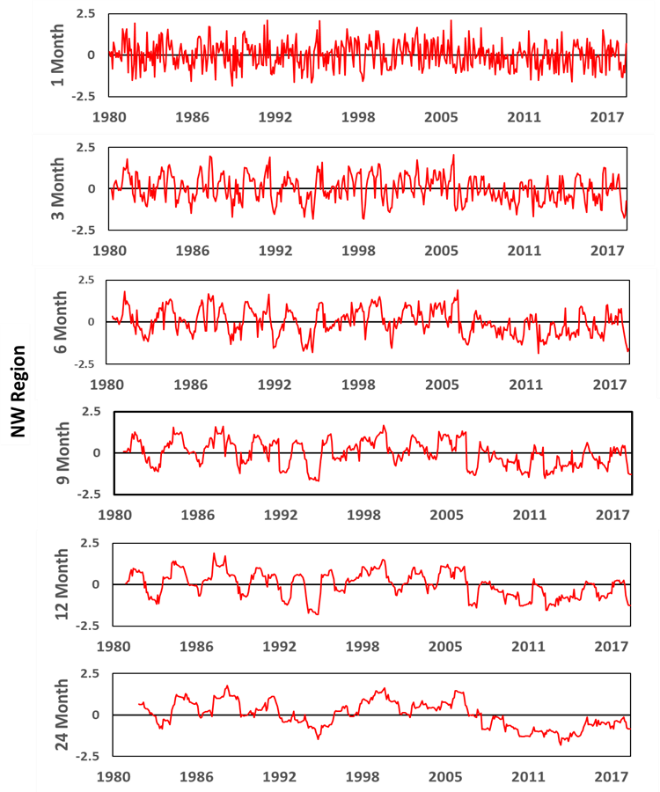** | **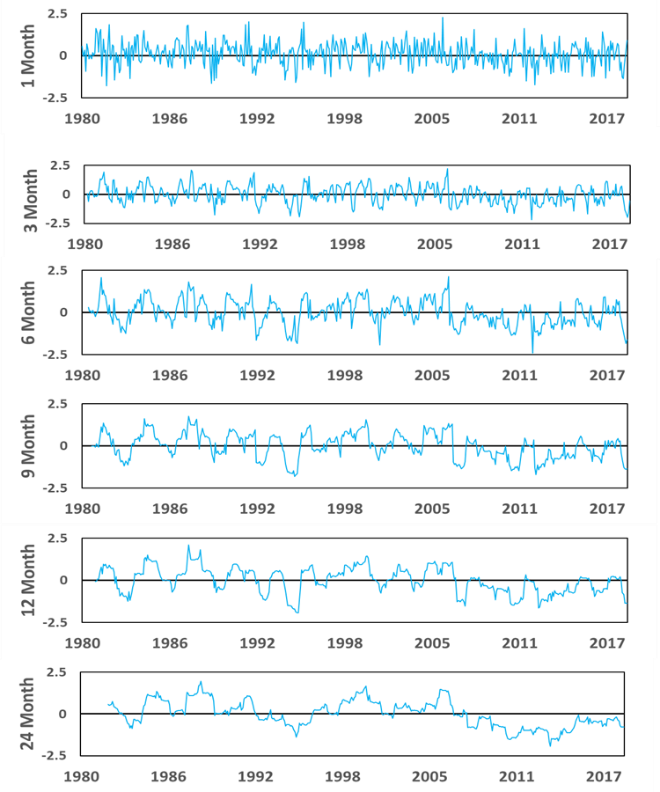** |
| --- | --- |

**Figure S1 (e) Same as Figure S1 (a), but for Northwest (NW) region**

| **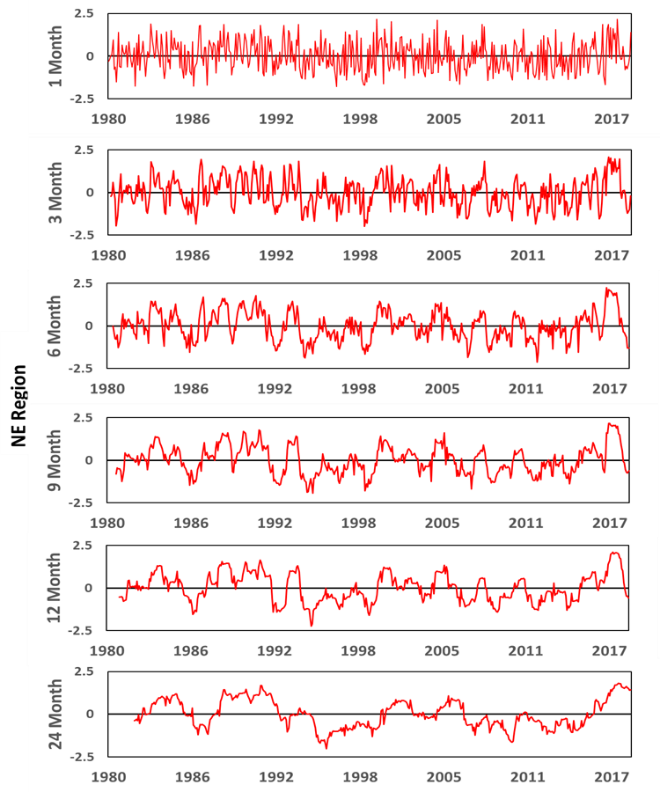** | **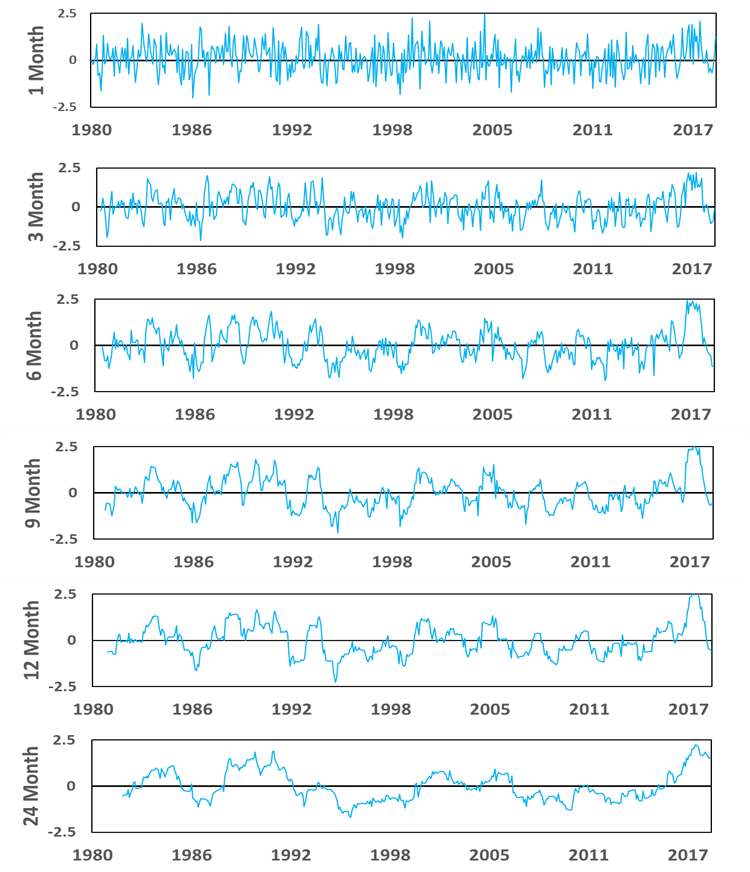** |
| --- | --- |

**Figure S1 (f) Same as Figure S1 (a), but for Northeast (NE) region**

| **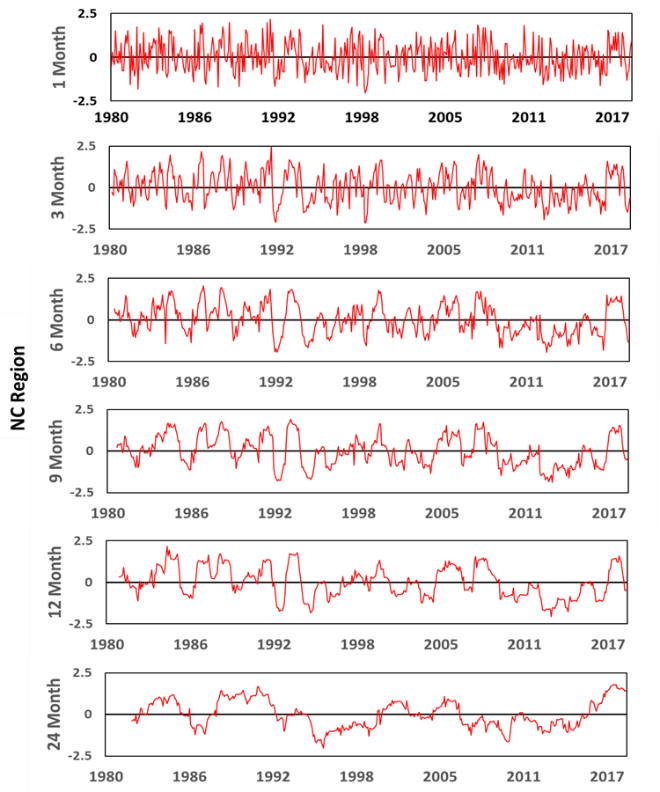** | **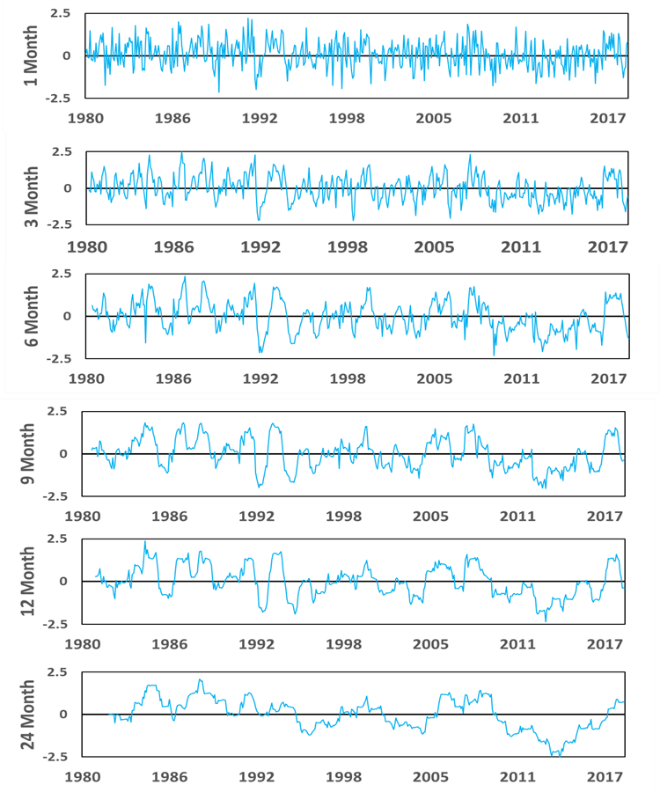** |
| --- | --- |

**Figure S1 (g) same as Figure S1 (a), but for North Central (NC) region**
